# Nocturnal blood pressure surge in seconds is a new determinant of left ventricular mass index

**DOI:** 10.1111/jch.14383

**Published:** 2021-12-21

**Authors:** Ayako Kokubo, Mitsuo Kuwabara, Yuki Ota, Naoko Tomitani, Shingo Yamashita, Toshikazu Shiga, Kazuomi Kario

**Affiliations:** ^1^ Division of Cardiovascular Medicine Department of Medicine Jichi Medical University School of Medicine Tochigi Japan; ^2^ Omron Healthcare Co., Ltd. Kyoto Japan

**Keywords:** beat‐by‐beat blood pressure monitor, blood pressure surge in seconds, blood pressure variability, nocturnal blood pressure

## Abstract

Nocturnal blood pressure (BP) surge in seconds (sec‐surge), which is characterized as acute transient BP elevation over several tens of seconds, could be a predictor of target organ damage. However, it is not clear that the severity of sec‐surge is different between sec‐surges induced by sleep apnea (SA) (apnea/hypopnea detected by polysomnography (PSG) or oxygen desaturation) and those induced by non‐SA factors (rapid eye movement, micro arousal, etc.), and sec‐surge variables associate with left ventricular hypertrophy (LVH) independently of conventional BP variables. The authors assessed these points with 41 patients (mean age 63.2±12.6 years, 29% female) who underwent full PSG, beat‐by‐beat (BbB) BP, and cuff‐oscillometric BP measurement during the night. All patients were included for the analysis comparing sec‐surge severity between inducing factors (SA and non‐SA factors). There were no significant differences in the number of sec‐surges/night between SA‐related sec‐surges and non‐SA‐related sec‐surges (19.5±26.0 vs. 16.4±29.8 events/night). There were also no significant differences in the peak of sec‐surges, defined as the maximum systolic BPs (SBPs) in each sec‐surge, between SA‐related sec‐surges and non‐SA‐related sec‐surges (148.2±18.5 vs. 149.3±19.2 mm Hg). Furthermore, as a result of multiple regression analysis (*n* = 18), the peak of sec‐surge was significantly and strongly associated with the left ventricular mass index (standardized β = 0.62, *p *= .02), compared with the mean nocturnal SBPs measured by oscillometric method (β = −0.04, *p* = .87). This study suggests that peak of sec‐surge could be a better predictor of LVH compared to parameters derived from regular nocturnal oscillometric SBP.

## 1 INTRODUCTION

Management of nocturnal blood pressure (BP) is one of the important issue for preventing the progress of hypertensive target organ damage (TOD) and the onset of cardiovascular disease.^1^, ^2^, ^3^ Recent studies have demonstrated that the nocturnal BP measured by ambulatory BP monitoring (ABPM) is a stronger predictor of cardiovascular events (CVE) than daytime BP.^4^, ^5^, ^6^, ^7^, ^8^, ^9^ Although ABPM has been the gold standard for assessing nocturnal BP, nocturnal BP measured by home BP monitoring (HBPM) also has high risk of future CVE^10^, ^11^, ^12^, ^13^, ^14^ and correlates with TOD as with ABPM.^15^ A new wrist‐type nocturnal HBPM device that automatically measures BP in the supine position without reducing sleep quality has been developed, and it could be used to facilitate real‐world sleeping studies.^16^, ^17^ In addition, the concern of an association between short‐term BP variability (BPV) and cardiovascular disease has been growing.^18^ Nocturnal BPV, defined as the standard deviation (SD) of nocturnal BP measured by ABPM, is associated with risks of CVE.^19^

It is assumed that some pathological factors such as obstructive sleep apnea (SA), rapid eye movement (REM) sleep, and microarousal increase nocturnal BPV.^20^, ^21^, ^22^ In particular, obstructive SA (OSA) has demonstrated a critical impact on nocturnal BP level and BPV.^23^ We have developed an oxygen‐triggered BP monitor and demonstrated that the maximum value of systolic BP (SBP) measured by the oxygen‐trigger function is higher than mean nocturnal SBP measured by the intermittent oscillometric method.^24^, ^25^, ^26^, ^27^, ^28^ However, as this BP monitor is based on the oscillometric method, the peak of short‐term BPV might be underestimated.

Consequently, we developed a continuous Beat‐by‐Beat (BbB) BP monitoring device using a tonometry method to detect the peak of the short‐term BPV in detail^29^. Using this device, we have already observed “BP surge in seconds (sec‐surge)”, which characterized as an acute transient BP elevation over several tens of seconds in OSA patients.^2^, ^30^ We have developed the tools for studies on sec‐surges such as the device and an automatic sec‐surge detection algorithm from BbB BP recordings overnight.^31^, ^32^ However, it is not clear whether the severity of sec‐surge is different between sec‐surges induced by SA (apnea/hypopnea detected by polysomnography (PSG) or oxygen desaturation) and those induced by non‐SA factors (sympathetic nerve activity such as REM sleep, micro arousal), and the CVE risks of sec‐surges.

Thus, this study aimed to assess the severity of the sec‐surge between inducing factors and to assess the association between left ventricular hypertrophy (LVH) and sec‐surge measured in a sleep laboratory setting.

## 2 METHODS

### 2.1 Study design and patients

In total, 48 outpatients were recruited for this study at the Washiya Hospital, Tochigi, Japan, from July, 2017 to February, 2019. Patients who met at least one of the following criteria were temporarily registered: (1) hypertensive patients, and (2) patients who had subjective symptoms of sleep apnea syndrome. Next, patients whose mean nocturnal SBP was ≥120 mm Hg, measured by ABPM or using a home BP monitor measuring BP automatically and intermittently, before 1 month of formal registration for the study were enrolled in this study. All registered patients underwent overnight full PSG, BP measurement using a BbB BP monitor worn on patient's left wrist and cuff‐oscillometric BP monitor worn on the patient's right arm for two nights within a month. After wearing these devices given them by study staff members, they were requested to sleep overnight at the sleep laboratory. A total of seven patients who had atrial fibrillation, missing data of PSG/BbB BP measurement in both nights, did not have adequate length of BbB BP records in both nights, or did not have sec‐surges in both nights were excluded from the study. In the end, 41 patients were included as study patients for assessing the difference in sec‐surge severity between sec‐surges induced by SA (apnea/hypopnea detected by polysomnography (PSG) or oxygen desaturation) and those induced by non‐SA factors (sympathetic nerve activity such as REM sleep, micro arousal). Eighteen patients who had inspection results of cardiac magnetic resonance imaging (MRI) within a year out of the 41 were included as study patients for assessing the association between sec‐surges and LVH. The detailed flow of study patients was shown in Figure S1.

The PSG was recorded by PSG‐1100 (Nihon‐Kohden) and analyzed by Polysmith (Nihon‐Kohden). The cardiac MRI was performed by OPTIMA MR450w Expert 1.5T (GE Healthcare). The measurement of left ventricular mass (LVM) was analyzed by cardiacVX (GE Healthcare) and Vitrea (Canon medical systems Corp) following our recent study.^33^ The LVM index (LVMI) was calculated as LVM/body surface area using the Fujimoto formula. The patients were recruited from Washiya Hospital, and this study was approved by the institutional review board of the Jichi Medical University School of Medicine, where this study was performed. Written informed consent was obtained from all patients upon their recruitment into the study.

### 2.2 Development of beat‐by‐beat BP monitoring device

**FIGURE 1:**
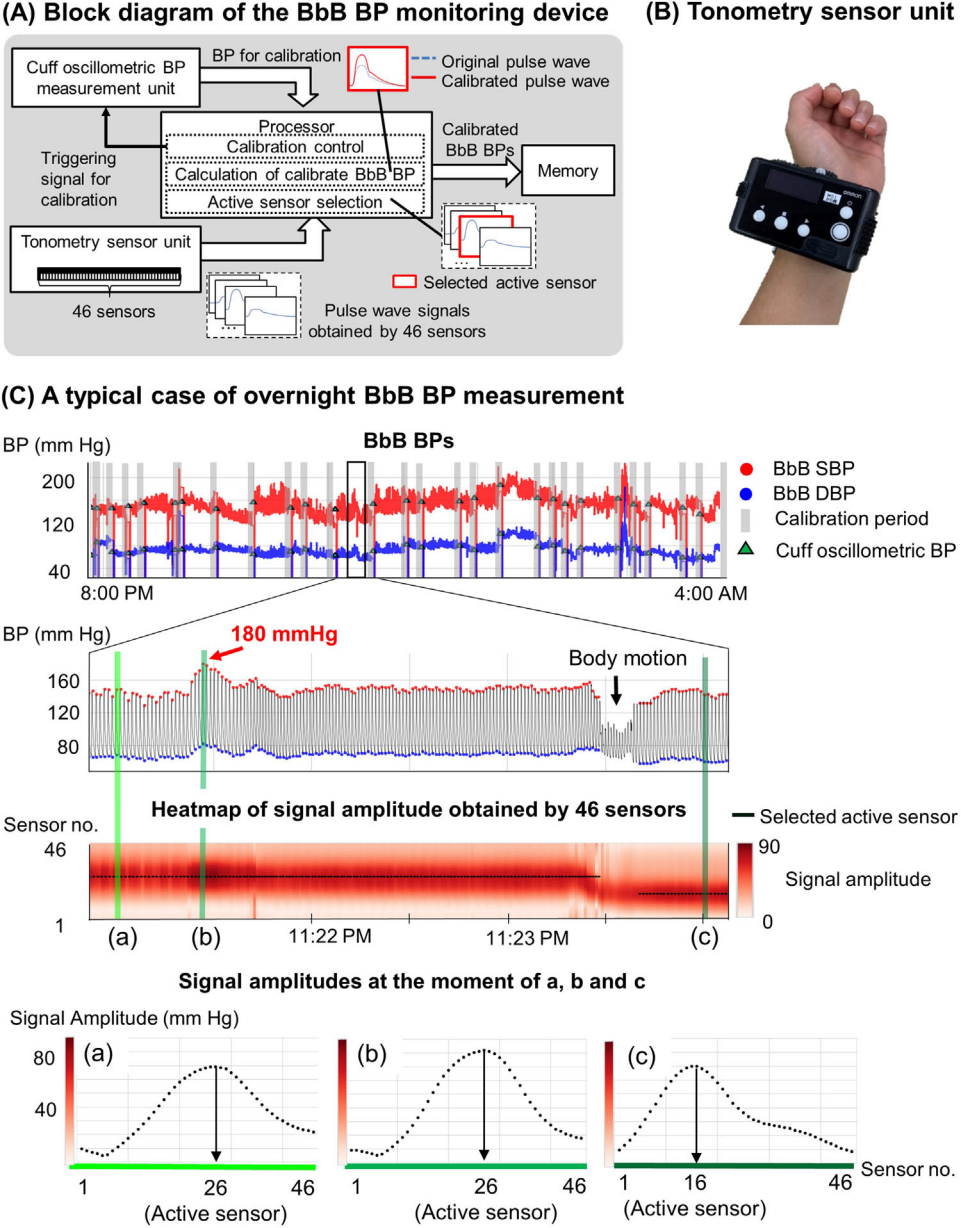
Overview of the BbB BP monitoring device. (A) A block diagram of the BbB BP monitoring device. (B) Tonometry sensor unit in the BbB BP monitoring device. (C) A typical case of overnight BbB BP measurement. BbB BPs were derived by the pulse wave signals at an active sensor and calibrated using cuff‐oscillometric BP. The active sensor is automatically selected based on the maximum amplitude of the pulse wave signals among 46 sensors at each moment. The heatmap indicates the amplitude of pulse wave signals obtained by 46 sensors as a colormap in a time series. (a), (b), and (c) show the amplitudes of pulse signals obtained by 46 sensors at each moment before the sec‐surge, peak of sec‐surge, and after changing the active sensor, respectively. BbB indicates beat‐by‐beat; BP, blood pressure; and sec‐surge, blood pressure surge in seconds

In the present study, overnight BbB BP was recorded by using the BbB BP monitoring device, based on the tonometry method^34^ we recently developed. Figure 1(A) shows a block diagram of the device. Pulse wave signals were obtained by 46 sensors in the “tonometry sensor unit^35^” (the appearance is shown in Figure 1(B)) directly placed on the skin above a radial artery, and were transmitted to the processor. Meanwhile, BP for calibration was measured when the “cuff‐oscillometric BP measurement unit” received a triggering signal from the “calibration control” function in the processor. The timing of calibration was automatically judged by the function when contact between the tonometry sensor and the skin was significantly changed due to body motion. Once the processor received BP for calibration, the “calculation of calibrated BbB BP” function transforms an amplitude of pulse wave signal obtained at an active sensor into the calibrated BbB BPs using the values of BP for calibration. Calibrated BbB BPs were recorded to the memory of the device. The active sensor was automatically selected based on the maximum amplitude of the pulse wave signal among 46 sensors at each moment in the “active sensor selection” function. The details are shown in Figure 1(C). The heatmap indicates the amplitude of pulse wave signals obtained by 46 sensors as a colormap in timeseries. The moment of (a), (b), and (c) show the signal amplitudes obtained by 46 sensors before the sec‐surge, peak of sec‐surge, and after changing the active sensor, respectively. The selected active sensor was kept as 26 in the stable section including both (a) and (b), although the signal amplitudes were increased at (b) due to sec‐surge. Even though the active sensor was changed from 26 to 16 by body motion, the maximum of signal amplitude and BbB BPs at (c) were maintained as (a). By selecting the active sensor automatically at each moment, the device could continuously monitor BbB BP without BP calibration, even though the contact between the tonometry sensor and the skin was slightly changed.

### 2.3 Definition of oscillometric BP variables

The office BP of the study patients was measured when they were recruited into this study. Conventional nocturnal BPs were measured using the arm‐cuff‐oscillometric BP monitor (HEM‐7220; Omron Healthcare Co., Ltd), which measures both intermittent BPs (30 min intervals) and oxygen‐triggered BPs. Oxygen‐triggered BPs were measured when the oxygen saturation (continuously monitored by pulse oximetry) falls below a variable threshold.^24^, ^25^, ^26^, ^27^ For further details of the definition of oscillometric BP variables, see Expanded Methods in the data supplement.

### 2.4 Definition of nocturnal beat‐by‐beat blood pressure surge in seconds (sec‐surge)

The sec‐surges were detected from overnight BbB BP recordings by an automatic sec‐surge detection algorithm^32^ we recently developed. For the detailed flow of detecting sec‐surge, see Expanded Methods in the data supplement.

**FIGURE 2:**
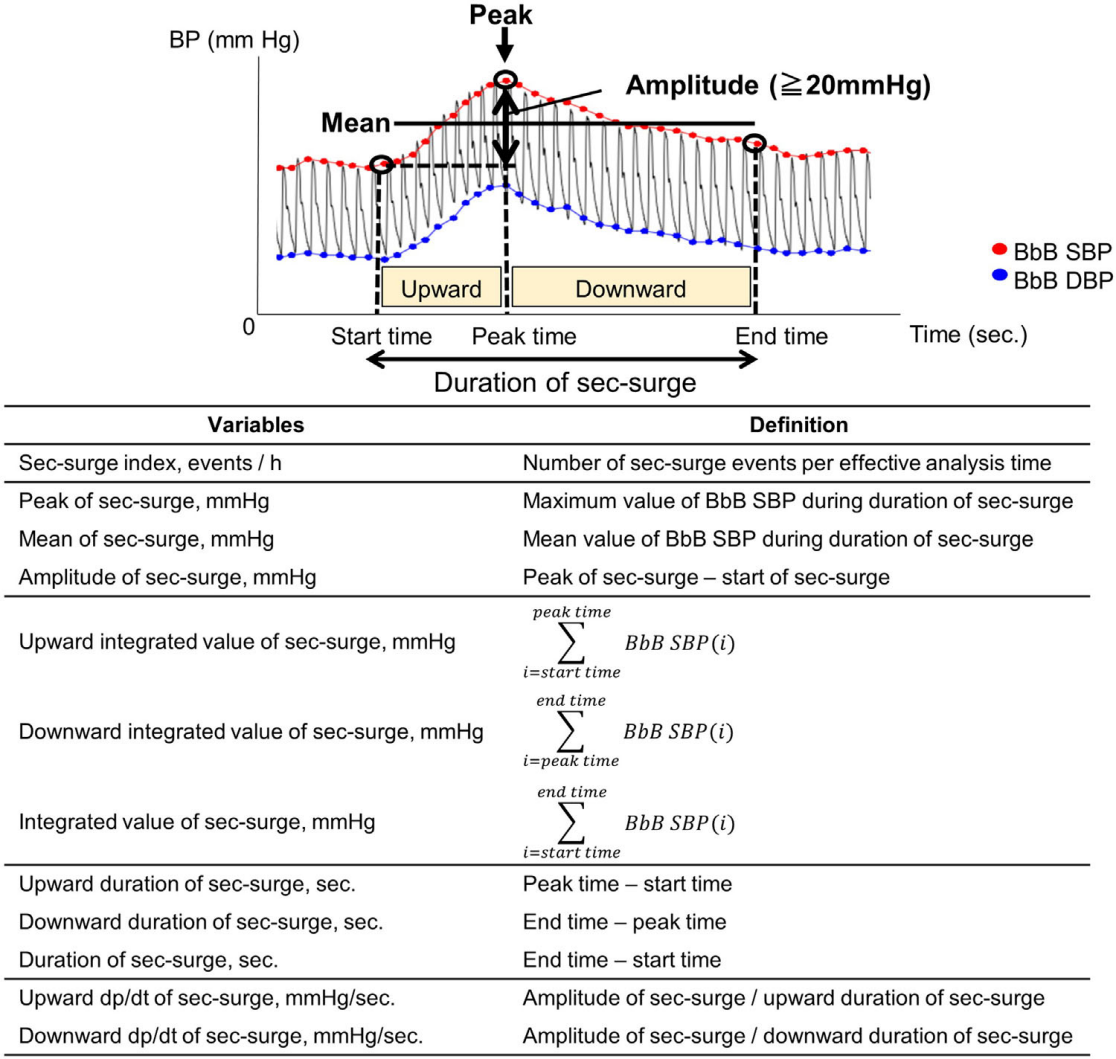
Definition of nocturnal blood pressure surge in seconds (sec‐surge) variables. BbB indicates beat‐by‐beat; SBP, systolic blood pressure; and DBP, diastolic blood pressure

We defined the sec‐surge feature as shown in Figure 2. All sec‐surge variables were calculated from the BbB SBPs between the start and end points of sec‐surges (duration of sec‐surge). The peak, start, and end point of the sec‐surge were detected by the above‐mentioned algorithm. The threshold of the amplitude of sec‐surge (difference between the peak SBP and the start SBP) was ≥20 mm Hg. The peak point was detected as the local maximum of BbB SBPs by using a sliding window. The start point was detected as the final point during stable BbB SBPs in backward‐searching ranges set before the peak point. The end point was detected as the point that SBP decreased by 75% of the amplitude of sec‐surge. We defined the duration from start to peak as upward duration and from peak to end as downward duration. The peak of sec‐surge and mean of sec‐surge were defined as the maximum value of BbB SBP and the mean value of BbB SBP, respectively, during duration of sec‐surge. The integrated values were calculated in upward, downward, and sec‐surge durations as the sum of BbB SBPs in each duration. The dp/dt of the sec‐surge was calculated in upward and downward durations as amplitude divided by the duration. Sec‐surge index was defined as number of sec‐surge events per effective analysis time. Each sec‐surge variable was taken as an average of the detected sec‐surges during the night. Furthermore, the mean, maximum, SD, coefficient of variation (CV), and average real variability (ARV) of nocturnal BbB SBPs were calculated from reliable BbB SBPs during the night. The maximum nocturnal BbB SBP was defined as the 95^th^ percentile of BbB SBP (instead of the maximum value) to avoid noises. The mean and maximum value of cuff‐oscillometric BPs used for BbB BP calibration (it was different from the abovementioned conventional nocturnal BP variables) were also calculated.

### 2.5 Labeling of sleep apnea and sleep stages to each sec‐surge

To investigate the sec‐surge features between sleep stages and between the inducing factors of sec‐surges (SA‐related sec‐surges and non‐SA‐related sec‐surges), labeling of sleep stages and sleep apnea for each sec‐surge was conducted. Sleep stages, apnea, and hypopnea were automatically determined by the Polysmith. For further details of the labeling, see Expanded Methods in the data supplement.

### 2.6 Statistical analysis

BP variables and PSG‐derived variables were calculated for each night. The average values of the two nights were used in the analysis. Continuous variables were expressed as mean±SD, and categorical variables were summarized as frequencies and percentages. In the analysis of sec‐surge severity between inducing factors of sec‐surges, Student's t‐test was used to compare SA‐related sec‐surges and non‐SA‐related sec‐surges in each sleep stage, and to compare nocturnal BP variables in severe OSA patients (AHI ≥ 30) and non‐severe OSA patients (AHI < 30). In the analysis of the associations between sec‐surge and LVH, correlations between LVMI and BP variables both sec‐surge and conventional one were analyzed using Pearson's correlation coefficient. Furthermore, to investigate the contribution of sec‐surge variables, multiple regression analyses were performed using a sec‐surge variable and a conventional BP variable as independent variables. The partial regression coefficient β were compared after calculating z‐score. Values of *p *< .05 in all statistical analyses were considered statistically significant. All statistical analyses were performed with R version 3.6.0.

## 3 RESULTS

The clinical characteristics of the study patients are shown in Table 1. In the analysis of the difference in sec‐surge severity between sec‐surges induced by SA (apnea or hypopnea) and those induced by non‐SA factors (sympathetic nerve activity), two of the 41 patients were normal in the perspective of apnea severity (AHI < 5), eight had mild OSA (5≤AHI < 15), seven had moderate OSA (15≤AHI < 30), and 24 had severe OSA (AHI ≥ 30). Table 2 shows intermittent BPs and oxygen‐triggered BPs measured by the cuff‐oscillometric method, and sec‐surge variables in each sleep stage. The peak of sec‐surge was ≥ 20 mm Hg higher than the mean of nocturnal SBPs measured by intermittent oscillometric method (127.2 ± 14.7 mm Hg and 148.2 ± 18.5 mm Hg). A typical case of sec‐surges is shown in Figure S4. Three sec‐surges were induced repeatedly, and the peak of sec‐surge reached almost 200 mm Hg from the baseline of 150 mm Hg. The distribution of the number of sec‐surges per patient is shown in Figure S5. In the analysis of associations between sec‐surges and LVH, the mean ± SD of LVMI in 18 patients who had LVM data was 68.9 ± 13.2 g/m^2^, and non‐severe LVH.

**TABLE 1:** Clinical characteristics of the study patients

	Study patients for analyzing sec‐surge severity between inducing factors (SA and non‐SA)			
	All (*n* = 41)	AHI < 30 (*n* = 17)	AHI ≥ 30 (*n* = 24)	Study patients for analyzing the association between sec‐surges and LVH (*n* = 18)
Age (years)	63.2 ± 12.6	60.5 ± 14.9	65.2 ± 10.6	65.0 ± 12.5
Female, *n* (%)	12 (29.3)	7 (41.2)	5 (20.8)	6 (33.3)
Body mass index (kg/m^2^)	27.3 ± 4.4	26.7 ± 4.8	27.7 ± 4.2	27.0 ± 4.6
Hypertension treatment, *n* (%)	33 (80.5)	14 (82.4)	19 (79.2)	15 (83.3)
Diabetes, *n* (%)	6 (14.6)	4 (23.5)	2 (8.3)	2 (11.1)
History of angina, *n* (%)	2 (4.9)	1 (5.9)	1 (4.2)	2 (11.1)
History of myocardial infarction, *n* (%)	1 (2.5)	0 (0.0)	1 (4.2)	1 (5.6)
History of stroke, *n* (%)	3 (7.3)	2 (11.8)	1 (4.2)	1 (5.6)
AHI, events/h	33.8 ± 21.0	14.0 ± 8.0	47.8 ± 15.0*	30.9 ± 24.4
Arousal index, events/h	22.9 ± 16.3	14.2 ± 4.2	29.2 ± 18.8*	18.1 ± 10.4
SpO2 < 90 %	19.6 ± 17.4	10.0 ± 11.9	26.3 ± 17.7*	16.3 ± 19.2
Lowest SpO2, %	77.7 ± 8.8	81.7 ± 7.0	74.9 ± 9.0*	79.3 ± 9.4
Total sleep time (min)	423.6 ± 93.7	446.2 ± 83.8	407.6 ± 98.7	419.1 ± 99.4
Sleep efficacy, %	68.5 ± 16.0	71.9 ± 14.5	66.1 ± 16.9	68.6 ± 17.4
REM, %	11.4 ± 6.0	11.3 ± 5.7	11.5 ± 6.3	12.1 ± 6.1
Non‐REM1, %	25.4 ± 11.6	17.9 ± 9.0	30.7 ± 10.3*	20.5 ± 9.7
Non‐REM2, %	57.1 ± 11.7	64.1 ± 9.0	52.1 ± 10.9*	60.7 ± 11.9
SWS, %	6.1 ± 7.5	6.8 ± 7.7	5.7 ± 7.5	6.7 ± 7.1

Data are expressed as mean ± SD or frequency and percentage.

*Abbreviations*: AHI, apnea hypopnea index; SpO2, oxygen saturation; REM, rapid eye movement; SWS, slow wave sleep.

* *P* < 0.05 versus patients with non‐severe obstructive sleep apnea (AHI < 30) using t‐test.

**TABLE 2:** Results of each nocturnal BP variable in each sleep stage (*n* = 41)

Nocturnal BP variables	Whole sleep period	Wake	REM	Non‐REM1	Non‐REM2	SWS
**Intermittent oscillometric BP**						
*Number of BP measurements*	16.7 ± 5.8	5.4 ± 3.9	1.2 ± 1.4	2.6 ± 1.7	6.9 ± 3.7	0.6 ± 0.8
SBP (mm Hg)	127.2 ± 14.7	129.9 ± 16.4	130.1 ± 18.7	127.5 ± 14.3	125.7 ± 15.4	116.1 ± 16.7
DBP (mm Hg)	77.6 ± 10.3	80.4 ± 12.0	79.6 ± 11.9	76.7 ± 11.1	76.8 ± 10.9	70.2 ± 9.7
PR (beats/min)	60.7 ± 8.9	63.3 ± 10.0	60.5 ± 9.9	60.6 ± 10.0	59.6 ± 8.2	59.2 ± 9.2
**Oxygen triggered oscillometric BP**						
*Number of BP measurements*	11.5 ± 14.2	2.2 ± 3.4	1.9 ± 3.4	2.9 ± 4.2	4.1 ± 6.6	0.3 ± 1.6
Hypoxia‐peak SBP (mm Hg)^a^	147.6 ± 22.7	–	–	–	–	–
Hypoxia‐mean SBP (mm Hg)	130.4 ± 18.5	128.6 ± 16.8	129.3 ± 19.9	127.5 ± 21.0	127.1 ± 20.7	110.6 ± 9.3
Hypoxia‐mean DBP (mm Hg)	79.7 ± 16.0	79.6 ± 16.6	78.6 ± 14.7	78.4 ± 12.3	78.4 ± 13.5	69.1 ± 10.9
Hypoxia‐mean PR (beats / min)	63.4 ± 9.1	64.5 ± 9.5	61.8 ± 11.1	61.9 ± 9.6	62.1 ± 9.9	61.7 ± 4.7
**Sec‐surge** ^b^						
Number of sec‐surges						
All, events	36.0 ± 40.2	5.4 ± 8.2	2.8 ± 4.0	6.0 ± 7.6	19.5 ± 23.3	2.2 ± 6.6
Induced by SA, events	19.5 ± 26.0	3.3 ± 6.4	1.6 ± 2.4	4.1 ± 6.4*	10.1 ± 14.7	0.4 ± 0.7
Induced by non‐SA factors, events	16.4 ± 29.8	2.1 ± 3.7	1.2 ± 2.7	1.9 ± 2.5	9.4 ± 17.7	1.9 ± 6.4
Peak of sec‐surge						
All (mm Hg)	148.2 ± 18.5	149.5 ± 19.6	151.2 ± 19.4	147.3 ± 19.7	147.4 ± 18.9	140.1 ± 16.7
Induced by SA (mm Hg)	148.2 ± 18.5	147.6 ± 20.0	151.3 ± 22.2	147.7 ± 21.4	147.3 ± 19.1	139.8 ± 15.5
Induced by non‐SA factors (mm Hg)	149.3 ± 19.2	151.1 ± 19.7	150.5 ± 24.4	145.8 ± 19.8	148.3 ± 20.0	139.7 ± 18.3
Amplitude of sec‐surge						
All (mm Hg)	25.8 ± 4.8	26.2 ± 6.0	26.0 ± 4.4	25.6 ± 3.5	25.7 ± 5.5	24.0 ± 3.6
Induced by SA (mm Hg)	26.0 ± 4.3	25.4 ± 7.0	27.0 ± 7.2	26.7 ± 5.0*	25.9 ± 4.4	25.3 ± 3.2
Induced by non‐SA factors (mm Hg)	25.8 ± 5.7	26.8 ± 6.5	25.5 ± 5.6	24.1 ± 3.0	25.6 ± 6.1	23.8 ± 4.1

Data are expressed as mean ± SD. SBP indicates systolic blood pressure; DBP, diastolic blood pressure.

*Abbreviations*: PR, pulse rate; REM, rapid eye movement; SWS, slow wave sleep; sec‐surge, blood pressure surge in seconds; SA, sleep apnea.

^a^ Hypoxia‐peak SBP was the highest SBP measured by oxygen‐triggered function.

^b^ Five out of 41 patients did not have any sec‐surges induced by SA, and another five patients did not have any sec‐surges induced by non‐SA factors. SA factors include apnea/hypopnea detected by polysomnography or oxygen desaturation. Non‐SA factors include sympathetic nerve activity such as REM sleep and micro arousal.

* *P* < 0.05 versus non‐SA‐related sec‐surges using t‐test.

### 3.1 Comparison of sec‐surge features between inducing factors

There was no significant difference in the peak of sec‐surges between SA‐related sec‐surges and non‐SA‐related sec‐surges in the whole sleep period (148.2±18.5 vs. 149.3±19.2 mm Hg) and each sleep stage (Table 2). Similarly, there were no significant differences in the number and amplitude of sec‐surges between SA‐related sec‐surges and non‐SA‐related sec‐surges (19.5±26.0 vs. 16.4±29.8 events/night and 26.0±4.3 vs. 25.8±5.7 mm Hg, respectively), except for the non‐REM1 stage. The associations between SA‐related sec‐surges and non‐SA‐related sec‐surges are shown in Figure S6. Both the peak and amplitude of SA‐related sec‐surge were significantly and strongly associated with those of non‐SA‐related sec‐surges (r = 0.874, *p* < .01, *n* = 31 and r = 0.473, *p* < .01, *n* = 31, respectively). Table 3 shows the comparison of each nocturnal BP variable in the whole sleep period between severe OSA (AHI ≥ 30) patients and non‐severe OSA (AHI < 30) patients. The number of sec‐surges induced by the SA factor was significantly higher among severe OSA patients than among non‐severe OSA patients (10.7 ± 10.3 vs. 25.8 ± 31.6 events/night). In contrast, that induced by non‐SA factors was significantly higher among non‐severe OSA patients than among severe OSA patients (29.9 ± 42.7 vs. 6.9 ± 6.8 events/night). There were no significant differences in the peak and amplitude of sec‐surge between severe OSA patients and non‐severe OSA patients (148.8±14.6 vs. 147.8±21.1 mm Hg and 26.3±6.0 vs. 25.4±3.9 mm Hg, respectively).

**TABLE 3:** Comparison of each nocturnal BP variable between severe and non‐severe OSA patients (*n* = 41)

	Whole sleep period	
Nocturnal BP variables	AHI < 30 (*n* = 17)	AHI ≥ 30 (*n* = 24)
**Intermittent oscillometric BP**		
*N*	17.5 ± 6.1	16.1 ± 5.7
SBP (mm Hg)	127.2 ± 12.1	127.2 ± 16.4
DBP (mm Hg)	78.3 ± 7.0	77.2 ± 12.1
PR (beats / min)	58.4 ± 8.3	62.2 ± 9.1
**Oxygen triggered oscillometric BP**		
*N*	5.3 ± 7.6	15.9 ± 16.2**
SBP (mm Hg)	128.9 ± 14.1	131.2 ± 20.4
DBP (mm Hg)	76.0 ± 10.1	81.4 ± 18.0
PR (beats / min)	62.0 ± 8.7	64.0 ± 9.4
**Sec‐surge**		
Number of sec‐surges		
All, events	40.6 ± 47.7	32.7 ± 34.7
Induced by SA, events	10.7 ± 10.3	25.8 ± 31.6*, **
Induced by non‐SA factors, events	29.9 ± 42.7	6.9 ± 6.8**
Peak of sec‐surge		
All (mm Hg)	148.8 ± 14.6	147.8 ± 21.1
Induced by SA (mm Hg)	149.4 ± 16.7	147.6 ± 19.7
Induced by non‐SA factors (mm Hg)	149.8 ± 14.4	149.0 ± 22.7
Amplitude of sec‐surge		
All (mm Hg)	26.3 ± 6.0	25.4 ± 3.9
Induced by SA (mm Hg)	26.4 ± 3.9	25.8 ± 4.6
Induced by non‐SA factors (mm Hg)	26.4 ± 6.6	25.3 ± 5.0

Data are expressed as mean ± SD.

*Abbreviations*: SBP, systolic blood pressure; DBP, diastolic blood pressure; PR, pulse rate; REM, rapid eye movement; SWS, slow wave sleep; sec‐surge, blood pressure surge in seconds; SA, sleep apnea; AHI, apnea hypopnea index.

* *P* < 0.05 versus non‐SA‐related sec‐surges using t‐test.

** *P* < 0.05 versus patients with non‐severe obstructive SA (AHI < 30) using t‐test.

### 3.2 Association between nocturnal BP variables and LVH

The BP measurements and correlations between LVMI and each BP variable were shown in Table 4. The mean nocturnal SBP measured using the oscillometric method and the mean nocturnal BbB SBP were almost comparable, and were distributed around the threshold for sleep SBP (120 mm Hg).^36^, ^37^ The mean of the oscillometric SBP for BbB BP calibration, the maximum of that, and the mean of nocturnal BbB SBP were significantly and strongly correlated with LVMI (r = 0.614, *p* < .01, *n* = 18; r = 0.635, *p* < .01, *n* = 18; and r = 0.492, *p* = .038, *n* = 18, respectively). The maximum of peak of sec‐surge and the mean of that were also significantly and strongly correlated with LVMI (r = 0.579, *p* = .012, *n* = 18; r = 0.607, *p* < .01, *n* = 18). The mean of peak of sec‐surge was correlated with the mean of oscillometric SBP for BbB BP calibration (r = 0.870, *p* < .01, *n* = 41). Even though the sec‐surges were classified as SA‐related sec‐surges and non‐SA‐related sec‐surges, significant and strong correlations were observed between peak of sec‐surge and LVMI in both SA‐related and non‐SA‐related sec‐surges (r = 0.551, *p* = .041, *n* = 14 and r = 0.606, *p* = .017, *n* = 15, respectively). The integrated values calculated in sec‐surge duration and downward duration had a marginally significant correlation with LVMI (r = 0.401, *p* = .099, *n* = 18 and r = 0.407, *p* = .094, *n* = 18, respectively). Although hypoxia‐peak SBP measured by the oxygen‐triggered oscillometric method was also significantly correlated with LVMI (r = 0.602, *p* = .038, *n* = 12), six out of the 18 patients did not have oxygen‐triggered BP measurements. There were no significant correlations between other conventional BP variables and LVMI.

**TABLE 4:** Simple Pearson's correlations between LVMI and blood pressure variables in study patients for analyzing the association between sec‐surges and LVH (*n* = 18)

BP variables	No. of BP measurements per one patient (Mean ± SD)	Measurement (Mean ± SD)	r	*p* value
**Conventional oscillometric BP (*n* = 18)**				
Office SBP (mm Hg)	2.0 ± 0.0	136.1 ± 15.7	0.256	.305
Evening SBP (mm Hg)	2.9 ± 0.2	125.2 ± 10.8	0.240	.338
Mean of nocturnal SBPs (mm Hg)	17.2 ± 4.3	122.6 ± 10.6	0.267	.285
Maximum of nocturnal SBP (mm Hg)	17.2 ± 4.3	141.4 ± 15.7	0.136	.591
Minimum of nocturnal SBP (mm Hg)	17.2 ± 4.3	106.0 ± 10.7	0.179	.477
Mean of three highest nocturnal SBPs (mm Hg)	17.2 ± 4.3	137.0 ± 14.5	0.157	.533
SD of nocturnal SBPs (mm Hg)	17.2 ± 4.3	9.7 ± 3.2	−0.066	.794
CV of nocturnal SBPs, %	17.2 ± 4.3	7.9 ± 2.4	−0.156	.536
Average real variability of nocturnal SBPs (mm Hg)	17.2 ± 4.3	9.0 ± 2.8	0.179	.478
**Oxygen triggered oscillometric BP (*n* = 12)** ^a^				
Hypoxia‐mean SBP (mm Hg)	15.6 ± 18.2	127.7 ± 13.1	0.388	.212
Hypoxia‐peak SBP (mm Hg)	15.6 ± 18.2	141.2 ± 14.2	0.602	.038
Nocturnal SBP surge (mm Hg)	15.6 ± 18.2	19.6 ± 13.2	0.288	.364
Maximum value of SBP surge (mm Hg)	15.6 ± 18.2	38.8 ± 16.6	0.259	.415
**Beat‐by‐beat BP (*n* = 18)**				
Mean of oscillometric SBP for BbB BP calibration (mm Hg)	23.3 ± 10.6	136.4 ± 15.0	0.614	<.01
Maximum of oscillometric SBP for BbB BP calibration (mm Hg)	23.3 ± 10.6	158.9 ± 18.8	0.635	<.01
Mean of nocturnal BbB SBPs (mm Hg)	14570 ± 7965	122.4 ± 15.3	0.492	.038
Maximum of nocturnal BbB SBP (mm Hg)	14570 ± 7965	150.0 ± 23.5	0.280	.260
SD of nocturnal BbB SBPs (mm Hg)	14570 ± 7965	16.2 ± 8.2	−0.085	.737
CV of nocturnal BbB SBPs, %	14570 ± 7965	13.3 ± 6.0	−0.218	.384
Average real variability of nocturnal BbB SBPs (mm Hg)	14570 ± 7965	2.6 ± 1.2	−0.030	.904
Sec‐surge index, events / h	32.8 ± 40.3	6.9 ± 6.5	0.239	.340
Maximum of peak of sec‐surge (mm Hg)	32.8 ± 40.3	165.1 ± 27.8	0.579	.012
Peak of sec‐surge (mm Hg)^b^	32.8 ± 40.3	144.6 ± 17.0	0.607	< .01
Mean of sec‐surge (mm Hg)^b^	851 ± 1100	132.8 ± 15.4	0.616	< .01
Amplitude of sec‐surge (mm Hg)^b^	32.8 ± 40.3	25.8 ± 6.8	0.157	.533
Upward integrated value of sec‐surge (mm Hg)^b^	412 ± 477	1546 ± 459	0.286	.250
Downward integrated value of sec‐surge (mm Hg)^b^	472 ± 671	1818 ± 539	0.401	.099
Integrated value of sec‐surge (mm Hg)^b^	851 ± 1100	3220 ± 829	0.407	.094
Upward duration of sec‐surge, sec^b^	32.8 ± 40.3	10.7 ± 3.4	0.033	.896
Downward duration of sec‐surge, sec^b^	32.8 ± 40.3	13.0 ± 4.5	0.172	.495
Duration of sec‐surge, sec^b^	32.8 ± 40.3	23.7 ± 6.5	0.138	.585
Upward dp/dt of sec‐surge (mm Hg/sec)^b^	32.8 ± 40.3	2.9 ± 0.8	0.296	.233
Downward dp/dt of sec‐surge (mm Hg/sec)^b^	32.8 ± 40.3	2.1 ± 1.2	−0.247	.324

Data are expressed as mean ± SD.

*Abbreviations*: LVMI, left ventricular mass index; sec‐surge, surge blood pressure in seconds; SBP, systolic blood pressure; DBP, diastolic blood pressure; SD, standard deviation; CV, coefficient of variation.

^a^ Oxygen desaturation was not detected by an oxygen‐triggered BP monitor in six patients.

^b^ Each sec‐surge variable was taken as an average of sec‐surges during the night.

As a result of four multiple regression models of LVMI using two independent variables (a sec‐surge variable and a conventional BP variable), the peak of sec‐surges had a significant correlation with LVMI. The other variables in each model were: (1) mean of nocturnal SBPs, (2) mean of nocturnal BbB SBPs, (3) SD of nocturnal SBPs, and (4) SD of nocturnal BbB SBPs. The standardized β in model 1 were 0.626 (*p* = .018) and −0.040 (*p* = .868), in the order of peak of sec‐surge and mean of nocturnal SBPs. The β in model 2 were 0.663 (*p* = .103) and −0.067 (*p *= .863), in the order of peak of sec‐surge and mean of nocturnal BbB SBPs. The β in model 3 were 0.723 (*p* = .003) and −0.325 (*p* = .130), in the order of peak of sec‐surge and SD of nocturnal SBPs. The β in model 4 were 0.604 (*p* = .010) and −0.041 (*p* = .843), in the order of peak of sec‐surge and SD of nocturnal BbB SBPs. The peak of sec‐surge was significant, except for model 2, and contributed more to LVMI than the other independent variables. The correlations between peak of sec‐surge and conventional BP variables are shown in Table S1. Although the peak of sec‐surge strongly correlated with the mean of nocturnal BbB SBPs (r = 0.916, *p* < .01, *n* = 41), there was no multicollinearity between the peak of sec‐surge and other conventional BP variables.

## 4 DISCUSSION

This study was the first to assess the nocturnal sec‐surge quantitatively using a BbB BP monitoring device based on the tonometry method in patients with OSA. The high BP value caused by sec‐surge was missed by conventional cuff‐oscillometric intermittent measurement (peak of sec‐surge measured by BbB BP monitoring device was 148.2 mm Hg and mean of nocturnal SBP measured by cuff‐oscillometric BP device was 127.2 mm Hg). Moreover, our results showed that the peak of sec‐surge was significantly and strongly associated with LVMI compared with the mean or SD of nocturnal SBP. Furthermore, no significant difference in the severity of sec‐surge was found between sec‐surges induced by SA (apnea or hypopnea) and those induced by non‐SA factors (sympathetic nerve activity). These findings imply that the peak of sec‐surge is a better predictor of LVH than conventional BP parameters regardless of inducing factors of sec‐surges. Assessing sec‐surges could be important for nocturnal BP management.

The peak of sec‐surge was ≥ 20 mm Hg higher than the mean of nocturnal SBP measured using the conventional oscillometric method. The intermittent BP measurement was insufficient to assess the risk of BP elevation. Whereas, the hypoxia‐peak SBP (the maximum value of SBP in the night measured by the oxygen‐triggered function) was 147.6 mm Hg and comparable with the peak of sec‐surge detected using the BbB BP monitor. However, the oxygen‐triggered BP monitor does not always detect the peak of BP elevation^38^ because the BP monitor takes three BP measurements based on the cuff‐oscillometric method at 15‐second intervals after the pulse oximeter senses oxygen desaturation.^25^ Almeneessier and coworkers have assessed and estimated BbB BPs from pulse transit time (PTT) using electrocardiography (ECG) and finger photoplethysmography in each section before, during, and after OSA events in a sleep setting.^39^ The estimated SBP increased by ∼4 mm Hg in non‐REM and ∼7 mm Hg in REM after OSA events compared with SBP before OSA events. Although this increase may indicate the amplitude of sec‐surge (peak SBP–start SBP) in this study, it might be underestimated compared with that (25.8 mm Hg) calculated from calibrated BbB SBPs. The accuracy of the estimated SBP and DBP using PTT is affected by arterial elasticity and pre‐ejection period (the time interval between QRS on ECG and the beginning of ventricular ejection).^30^, ^40^ Especially, PTT calculated at a finger might affect the property of the artery more. Moreover, although we identified the peak and start points of sec‐surges using the algorithm^32^ for calculating the amplitude, they did not detect them accurately. At present, the method for assessing the peak of sec‐surge overnight is only using the BbB BP measurement method based on the tonometry technology and the automatic sec‐surge detection algorithm.

The peak of sec‐surge was strongly associated with LVMI independently of conventional mean nocturnal SBP measured using the oscillometric method every 30 min in the present study. To the best of our knowledge, no study has demonstrated the association between LVH and sec‐surges. Palatini and coworkers have demonstrated that general nocturnal BPV, defined as the SD of BP measured by ABPM every 15 or 30 min, is associated with the risk of CVEs using 7112 hypertensive patients in a prospective study.^19^ In this study, the contribution to LVMI was stronger in the peak of sec‐surges than the SD of nocturnal SBPs. In addition, no correlations were found between sec‐surge variables and SD, CV, and ARV of nocturnal SBPs measured using the cuff‐oscillometric method (Table S1). We are now proposing the “resonance hypothesis of BPV^31^”. BPV is induced in different time phases (yearly, seasonal, day‐by‐day, diurnal, and BbB). When the timing of all BPVs with different time phases is synchronized, it generates a critically large dynamic BP surge that would trigger a CVE. If the general nocturnal BPV defined as the SD of intermittent oscillometric BP is synchronized with the sec‐surge, the cardiac overload would be more critical. Additionally, the maximum of peak of sec‐surge and the mean of that were similarly correlated with LVMI (r = 0.579, *p* = .012, *n* = 18; and r = 0.607, *p* < .01, *n* = 18, respectively). We hypothesized that mean of peak of sec‐surge is suited to evaluate LVH because it may represent increased cardiac overload during the night. On the other hand, occurrence of CVE during the night may likely be triggered by the maximum of peak of sec‐surge.

Considering BbB BPV, no associations were observed between LVMI and nocturnal BbB BPV variables (SD, CV, and ARV), and the peak of sec‐surge was better for assessing LVMI than these variables in this study. Several studies have reported that daytime 5–10 min of BbB BPV measured using a Finometer (volume compensation method using finger cuff) in the supine position is a potential predictor of stroke or target organ damage. Webb and coworkers have demonstrated that 5‐min BbB BPV progressed with increasing age, and had a similar predictive power of recurrent stroke and CVEs compared with that of day‐to‐day BPV in patients with cerebrovascular events.^41^, ^42^ Furthermore, Wei and coworkers have demonstrated that 10‐min BbB BPV variables were associated with LVMI independently of the BP level (the effect sizes were +2.97–3.53 g/m^2^) in 128 untreated hypertensive patients.^43^ The BbB BP measurements in this study were collected under an overnight sleep condition and might have a different meaning from BbB BPV variables in previous studies.

Considering the level of nocturnal BP, although the mean of nocturnal SBP (measured using the cuff‐oscillometric method) was not significantly associated with LVMI (r = 0.267; *p* = .285; *n* = 18), the mean of nocturnal BbB SBP was significantly associated with LVMI (r = 0.492; *p* = .038; *n* = 18). The results implied that using a large sample of nocturnal BPs for calculating the BP level (∼14 570 measurements in this study) might assess LVH more accurately. Our previous Japan Morning Surge Home Blood Pressure (J‐HOP) study has demonstrated that the mean nocturnal SBP measured at 2:00 a.m., 3:00 a.m., and 4:00 a.m. is significantly associated with LVMI (r = 0.18; *p* < .001; *n* = 2563) in outpatients with one or more cardiovascular risks enrolled at multiple centers.^44^ The strength of the association between LVMI and the mean nocturnal SBP in this study conform to the results of the J‐HOP study. Furthermore, our results showed that the hypoxia‐peak SBP measured using an oxygen‐triggered BP monitor was significantly associated with LVMI (r = 0.602; *p* = .038; *n* = 12). However, the BP monitor only detects the sec‐surges induced by SA, and oxygen desaturation was actually not detected by the BP monitor in six out of 18 patients.

Surprisingly, no significant differences in the severity of sec‐surges (number, peak, and amplitude) were found between SA‐related sec‐surges induced by apnea/hypopnea or oxygen desaturation and non‐SA‐related sec‐surges induced by sympathetic nerve activity in patients with OSA. It is known that baroreceptor reflex sensitivity is depressed in patients with OSA.^45^ It is assumed that frequent non‐SA‐related sec‐surges were induced by sympathetic nerve activation due to the baroreflex dysfunction in patients with OSA. The average number of sec‐surges/night was 36, and 16 of 36 sec‐surges were induced by non‐SA factors. In other words, 44% of sec‐surges might not be assessed by the aforementioned oxygen‐triggered BP monitor. No study has reported that non‐SA‐related sec‐surges were induced ≥40% of sec‐surges that occurred during the night. Furthermore, both peak of SA‐related and non‐SA‐related sec‐surges were significantly and strongly correlated with LVMI (r = 0.551, *p* = .041, *n* = 14 and r = 0.606, *p* = .017, *n* = 15, respectively). The peak of sec‐surges induced by any factors (SA and non‐SA) might be a better predictor of LVH, and these sec‐surges might be more accurately detected and assessed using the BbB BP monitoring device than other BP measurement methods.

All significant sec‐surge variables for assessing LVMI resulted from absolute BP values, although we calculated various variables including the duration or dp/dt of sec‐surges. These results suggest that absolute BP values more directly reflected cardiac overload than other BP variables, including the amplitude of sec‐surges. During sleep, LV preload is augmented by the increase in venous return from the lower body to the heart due to the supine position.^2^ It is assumed that the further overload caused by sec‐surges under such an augmented preload condition brought about a strong association between sec‐surge and LVH.

The present study has some limitations. First, this study was conducted on a small sample composed of patients with OSA and focused on only nocturnal BP variables for the evaluation. Although sec‐surge variables were not directly compared with daytime one in this study, recent studies have demonstrated the nocturnal BP measured by ABPM is more associated with CVE than daytime BP. Sec‐surge may be a better predictor for CVE risk evaluation. Furthermore, despite the small sample size, we demonstrated a strong association between LVMI and the peak of sec‐surges owing to a large number of BbB BP measurements for BP evaluation. To verify the findings of this study, other studies including patients without SA and larger‐scale clinical trials are required. Second, the BbB BP calibration was triggered many times (approximately 23.3 times) during the night when BP changed by some factors such as repeated sec‐surge. In short, the calibration SBP might detect a part of phenomenon of the sec‐surge. The correlation coefficient between the peak of sec‐surge and the calibration SBP was 0.870, and both variables were strongly correlated with LVMI (r = 0.607, *p* < .01, *n* = 18; and r = 0.614, *p* < .01, *n* = 18, respectively). Detecting higher BP value by BbB BP monitoring with fewer calibration might be important in clinical evaluation. Third, a validation of our BbB BP monitoring device was not currently performed. A validation method for continuous BP monitoring has not yet been established by the International Organization for Standardization. We are now considering the following points for a new validation method: (1) accuracy of the level of BbB BP values are assessed using the accuracy of a BP monitor for calibration, and (2) accuracy of the amount of BPV induced by Valsalva maneuver is assessed by comparing it with a validated continuous BP monitoring device. Our recent study has demonstrated that the error between BbB BPs measured by our BbB BP monitoring device and those measured by a conventional BbB BP monitor (JENTOW‐7700, Nihon Colin, Japan) was −0.3 ± 4.7 mm Hg in SBP, and 0.7 ± 3.4 mm Hg in DBP, respectively.^29^ The study has been conducted in a controlled laboratory setting and evaluated BbB BPs measured in both stable state and BP elevation state induced by the Valsalva maneuver. In the present sleep setting study, although biological BP changing or pressure signal reduction due to contact state changing between the sensor and skin might be occurred, our BP monitoring device can be recalibrated for robust measurement when BP significantly changing from the previous calibration BP.

## 5 CONCLUSIONS AND PERSPECTIVES

The peak of sec‐surge measured using the BbB BP monitoring device was strongly associated with LVH and was better predictor of LVH than conventional nocturnal BP variables. Furthermore, there were no significant differences in the peak, amplitude, and number of sec‐surges between sec‐surges induced by SA (apnea or hypopnea) and those induced by non‐SA factors (sympathetic nerve activity). Sec‐surges brought about by nocturnal BP assessment using the BbB BP monitor have a potential of worth monitoring to the management of nocturnal BP for preventing LVH, regardless of the inducing factors of sec‐surges. Further studies of nocturnal sec‐surges or short‐term BPV will be needed to understand the mechanisms and implications because current knowledge about them is not widely studied. In addition, to facilitate future clinical study, the development of the BbB BP monitor with better usability and durability for overnight measurement is quite important. Patients with resistant hypertension tend to have nocturnal hypertension. In the present study, the sec‐surges occurred frequently in the night, and the peak of them were missed by the conventional cuff‐oscillometric measurement. The assessment and management of sec‐surges will potentially facilitate the understanding of the mechanisms of resistant hypertension.

## CONFLICT OF INTEREST

K Kario received research grants from Omron Healthcare, and is a consultant for Omron Healthcare. A Kokubo works for Omron Healthcare Co. Ltd and belongs to Jichi medical university as a graduate student. M Kuwabara and T Shiga work for Omron Healthcare Co. Ltd and are visiting researchers at the Jichi medical university.

## AUTHORS CONTRIBUTION

K. Kario supervised the conduct of the study and data analysis, and had the primary responsibility of writing this paper. A. Kokubo analyzed the data and wrote the Introduction, Methods, Results, and Discussion sections. M. Kuwabara, N. Tomitani, and S. Yamashita contributed to the analysis and reviewed the manuscript. Y. Ota wrote the Methods section. T. Shiga supervised the conduct of the study. A. Kokubo, M. Kuwabara, Y. Ota, and S. Yamashita collected the data. All authors discussed the results and contributed to the final manuscript.

## Supporting information

Supporting material
