# Supplementary material for: Nocturnal blood pressure surge in seconds is a new determinant of left ventricular mass index
Source: J Clin Hypertens (Greenwich). 2021 Dec 21;24(3):271–82. doi: 10.1111/jch.14383 (PMC8925009; doi:10.1111/jch.14383)
Supplement: Supplementary file 1 — Supporting material [file JCH-24-271-s001.docx]

Supplementary file

**Nocturnal Blood Pressure Surge in Seconds is a New Determinant of**

**Left Ventricular Mass Index**

Ayako Kokubo ME^1, 2^, Mitsuo Kuwabara PhD^1, 2^, Yuki Ota ME^2^, Naoko Tomitani BSc^1^,

Shingo Yamashita ME^2^, Toshikazu Shiga, PhD^1, 2^ and Kazuomi Kario MD, PhD^1^

^1^Division of Cardiovascular Medicine, Department of Medicine, Jichi Medical University School of Medicine, Tochigi, Japan

^2^Omron Healthcare Co., Ltd., Kyoto, Japan

**Address correspondence to:** Kazuomi Kario MD, PhD, Division of Cardiovascular Medicine, Department of Medicine, Jichi Medical University School of Medicine, 3311-1, Yakushiji, Shimotsuke, Tochigi 329-0948, Japan; e-mail: kkario@jichi.ac.jp

***Expanded Methods***

***Definition of oscillometric BP variables***

All conventional BPs are shown in supplemental Figure S2. The evening SBP was defined as the mean of 3 SBP values measured manually before going to sleep in the supine position. The mean, minimum, maximum, SD, and coefficient of variation (CV) of nocturnal SBPs were calculated from all intermittent SBPs. The mean of the three highest nocturnal SBPs was defined as the mean of the three highest intermittent SBP values. The average real variability (ARV) was defined as the average of the absolute differences of consecutive measurements^1^. The hypoxia-peak SBP and hypoxia-mean SBP were defined as the maximum and the mean SBP values, respectively, measured by the oxygen-triggered function. The nocturnal SBP surge was defined as the difference between the hypoxia-peak SBP and mean of nocturnal SBPs. The maximum value of the SBP surge was defined as the difference between the hypoxia-peak SBP and the minimum of nocturnal SBP^2^.

***Definition of Nocturnal beat-by-beat blood pressure surge in seconds (sec-surge)***

Firstly, low-reliability periods of overnight BbB BP recordings were excluded by the data cleansing function of the algorithm. The criteria for low-reliability were 1) body motion, 2) failure of pulse detection due to the measurement device, 3) outlier BP values, 4) large BP shifts from calibrated BP measured by oscillometric method, and 5) low signal amplitude from the tonometry sensor. Secondly, the candidates of sec-surges in each subject were screened by the algorithm. Thirdly, the candidates were manually classified as sec-surge, noise, or undetermined BPV following the visual check procedure^3^. The cases that were classified as sec-surge were used in this study.

***Labeling of sleep apnea and sleep stages to each sec-surge***

Sleep stages were automatically determined for each 30-second epoch. Apnea was defined as a ≥ 90% decrease in the airflow signal from baseline for ≥ 10 seconds. Hypopnea was defined as a ≥ 30% reduction in the airflow from baseline for ≥ 10 seconds, accompanied by a ≥ 4% reduction in oxygen saturation. These detections and the period of oxygen desaturation were determined by the Polysmith.

Sleep apnea for each sec-surge was labeled when the sec-surge met either of following conditions: (1) The peak of sec-surge existed in the period from the start of apnea/hypopnea to the end of it + X_1_ seconds, and (2) the peak of sec-surge existed in the period of oxygen desaturation and oxygen saturation falls by ≥ 4% from the baseline. The value of X_1_ was determined by the distribution of the upward duration of sec-surges using 95^th^ percentiles. BP elevation of the sec-surge starts at the timing of release of SA^4^. We assumed that the upward time of the sec-surge covered the time between the end of apnea/hypopnea and the peak of the sec-surge. Sec-surges that had the sleep apnea label were used as SA-related sec-surges, while those that did not have the label were used as non-SA-related sec-surges in the statistical analysis. The sleep stage for each sec-surge was labeled as the stage of the epoch in which the peak of the sec-surge existed. For example, if the peak of the sec-surge existed in the REM stage epoch, the label of sleep stage was set as REM. The details of labeling rules are shown in supplemental Figure S3. The value of X_1_, criteria for labeling SA to each sec-surge was 21.2 seconds.

***References:***

1. Hansen TW, Thijs L, Li Y, et al. Prognostic Value of Reading-to-Reading Blood Pressure Variability Over 24 Hours in 8938 Subjects From 11 Populations. *Hypertension*. 2010;55(4):1049-1057. doi:10.1161/HYPERTENSIONAHA.109.140798

2. Sasaki N, Nagai M, Mizuno H, Kuwabara M, Hoshide S, Kario K. Associations Between Characteristics of Obstructive Sleep Apnea and Nocturnal Blood Pressure Surge. *Hypertension*. 2018;72(5):1133-1140. doi:10.1161/HYPERTENSIONAHA.118.11794

3. Kokubo A, Kuwabara M, Nakajima H, et al. Automatic detection algorithm for establishing standard to identify “surge blood pressure.” *Med Biol Eng Comput*. 2020;58(6):1393-1404. doi:10.1007/S11517-020-02162-4

4. Somers VK, Dyken ME, Clary MP, Abboud FM. Sympathetic neural mechanisms in obstructive sleep apnea. *J Clin Invest*. 1995;96(4):1897-1904. doi:10.1172/JCI118235

| **Supplementary Table S1.** Correlations between sec-surge and conventional BPs (*n* = 41) | | | | | | | | | | | | | | | | | | |
| --- | --- | --- | --- | --- | --- | --- | --- | --- | --- | --- | --- | --- | --- | --- | --- | --- | --- | --- |
| **Correlate** | **Sec-surge** | | | | | | | | | | | | | | | | | |
|  | Sec-surge index | | Peak | | Mean | | Amplitude | | Upward integrated value | | Downward integrated value | Integrated value | Upward duration | Downward duration | Duration | Upward dp/dt | Downward dp/dt |  |
| **Conventional oscillometric BP** | | | | | | | | | | | | | | | | | | |
| Office SBP | -0.031 | | 0.226 | | 0.229 | | -0.004 | | 0.035 | | 0.083 | 0.067 | -0.051 | 0.028 | -0.005 | -0.006 | 0.032 |  |
| Evening SBP | -0.014 | | 0.552 † | | 0.564 † | | -0.018 | | 0.215 | | -0.119 | 0.022 | 0.001 | -0.316 * | -0.219 | 0.060 | 0.288 |  |
| Mean of nocturnal SBPs | -0.068 | | 0.640 † | | 0.632 † | | 0.181 | | 0.376 * | | -0.093 | 0.119 | 0.167 | -0.315 * | -0.139 | 0.040 | 0.267 |  |
| Maximum of nocturnal SBP | -0.075 | | 0.548 † | | 0.533 † | | 0.213 | | 0.324 * | | -0.175 | 0.042 | 0.168 | -0.376 * | -0.181 | 0.021 | 0.362 * |  |
| Minimum of nocturnal SBP | -0.064 | | 0.564 † | | 0.572 † | | 0.074 | | 0.302 | | -0.034 | 0.121 | 0.051 | -0.233 | -0.138 | 0.097 | 0.173 |  |
| Mean of 3 highest nocturnal SBPs | -0.092 | | 0.561 † | | 0.544 † | | 0.225 | | 0.324 * | | -0.179 | 0.040 | 0.182 | -0.366 * | -0.167 | 0.015 | 0.363 * |  |
| SD of nocturnal SBPs | -0.115 | | 0.109 | | 0.077 | | 0.190 | | 0.066 | | -0.289 | -0.153 | 0.168 | -0.299 | -0.128 | -0.112 | 0.325 * |  |
| CV of nocturnal SBPs | -0.109 | | -0.025 | | -0.058 | | 0.169 | | -0.014 | | -0.299 | -0.197 | 0.130 | -0.268 | -0.124 | -0.105 | 0.305 |  |
| ARV of nocturnal SBPs | -0.024 | | 0.119 | | 0.098 | | 0.026 | | 0.027 | | -0.262 | -0.156 | 0.104 | -0.267 | -0.136 | -0.148 | 0.257 |  |
| **Oxygen triggered oscillometric BP** | | | | | | | | | | | | | | | | | |  |
| Hypoxia-mean SBP | -0.018 | 0.584 † | | 0.572 † | | 0.209 | | 0.250 | | -0.255 | | -0.055 | 0.098 | -0.433 † | -0.272 | 0.087 | 0.344 |  |
| Hypoxia-peak SBP | -0.081 | 0.643 † | | 0.642 † | | 0.247 | | 0.455 † | | -0.127 | | 0.125 | 0.072 | -0.416 * | -0.271 | 0.160 | 0.356 * |  |
| Nocturnal SBP surge | -0.181 | 0.175 | | 0.171 | | 0.172 | | 0.273 | | -0.143 | | 0.036 | -0.019 | -0.287 | -0.218 | 0.144 | 0.274 |  |
| Maximum value of SBP surge | -0.123 | 0.249 | | 0.236 | | 0.182 | | 0.317 | | -0.199 | | 0.020 | 0.188 | -0.340 | -0.167 | -0.111 | 0.295 |  |
| **Beat-by-beat BP** | | | | | | | | | | | | | | | | | |  |
| Mean of nocturnal BbB SBPs | 0.095 | 0.916 † | | 0.924 † | | 0.146 | | 0.448 † | | 0.188 | | 0.329 * | -0.027 | -0.221 | -0.166 | 0.298 | 0.102 |  |
| Maximum of nocturnal BbB SBP | 0.026 | 0.758 † | | 0.772 † | | 0.081 | | 0.296 | | 0.066 | | 0.177 | -0.145 | -0.288 | -0.269 | 0.342 * | 0.167 |  |
| SD of nocturnal BbB SBPs | -0.089 | 0.105 | | 0.125 | | -0.110 | | -0.093 | | -0.186 | | -0.168 | -0.263 | -0.265 | -0.310 * | 0.201 | 0.181 |  |
| CV of nocturnal BbB SBPs | -0.122 | -0.156 | | -0.138 | | -0.168 | | -0.229 | | -0.286 | | -0.296 | -0.272 | -0.256 | -0.308 * | 0.121 | 0.210 |  |
| ARV of nocturnal BbB SBPs | -0.261 | 0.333 * | | 0.297 | | 0.455 † | | 0.017 | | -0.211 | | -0.133 | 0.012 | -0.230 | -0.154 | 0.205 | 0.315 * |  |
| All values are Pearson’s correlation coefficients. Sec-surge indicates blood pressure in seconds; SBP, systolic blood pressure; DBP, diastolic blood pressure; SD, standard deviation; and CV, coefficient of variation; and ARV, average real variability. * *P* < 0.05. †*P* < 0.01. | | | | | | | | | | | | | | | | | |  |


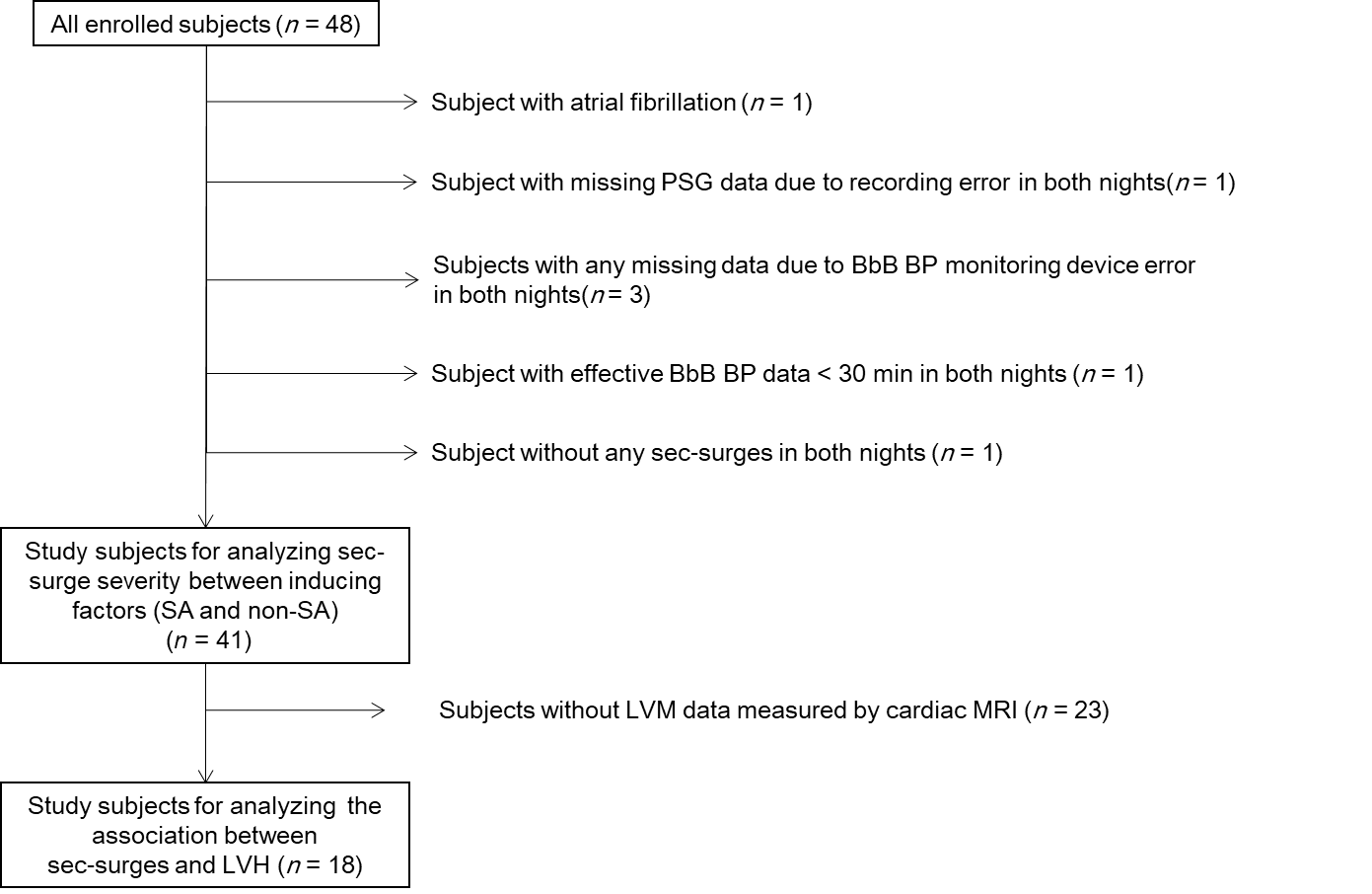


**Supplementary Figure S1.** Flow chart of study subject selection. BbB indicates beat-by-beat; BP, blood pressure; sec-surge, blood pressure surge in seconds; PSG, polysomnography; SA, sleep apnea; MRI, magnetic resonance imaging; LVM, left ventricular mass; and LVH: left ventricular hypertrophy.


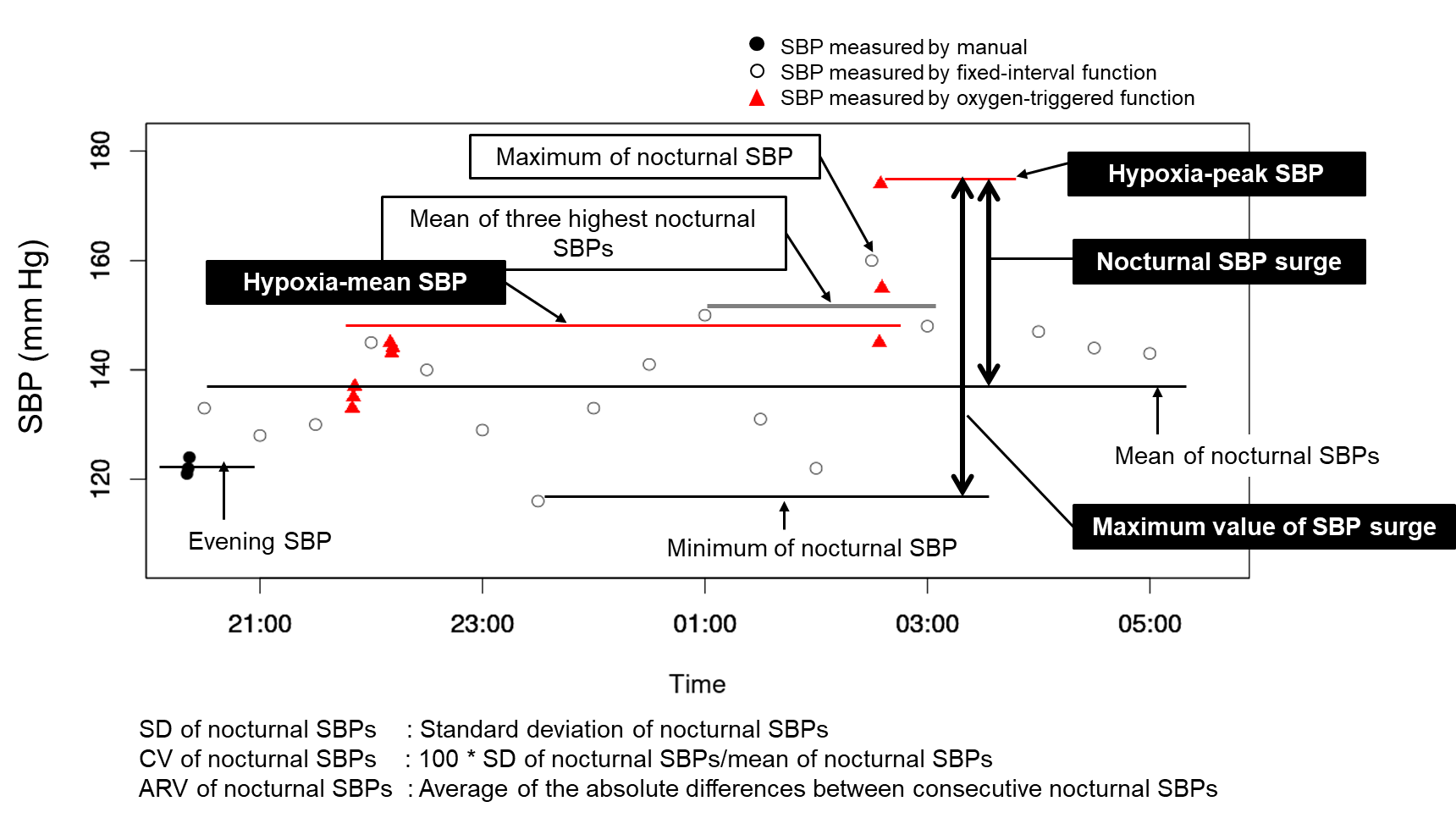


**Supplementary Figure S2.** Definition of nocturnal blood pressure variables measured by the cuff oscillometric method. SBP indicates systolic blood pressure; SD, standard deviation; CV, coefficient of variation; and ARV, average real variability.


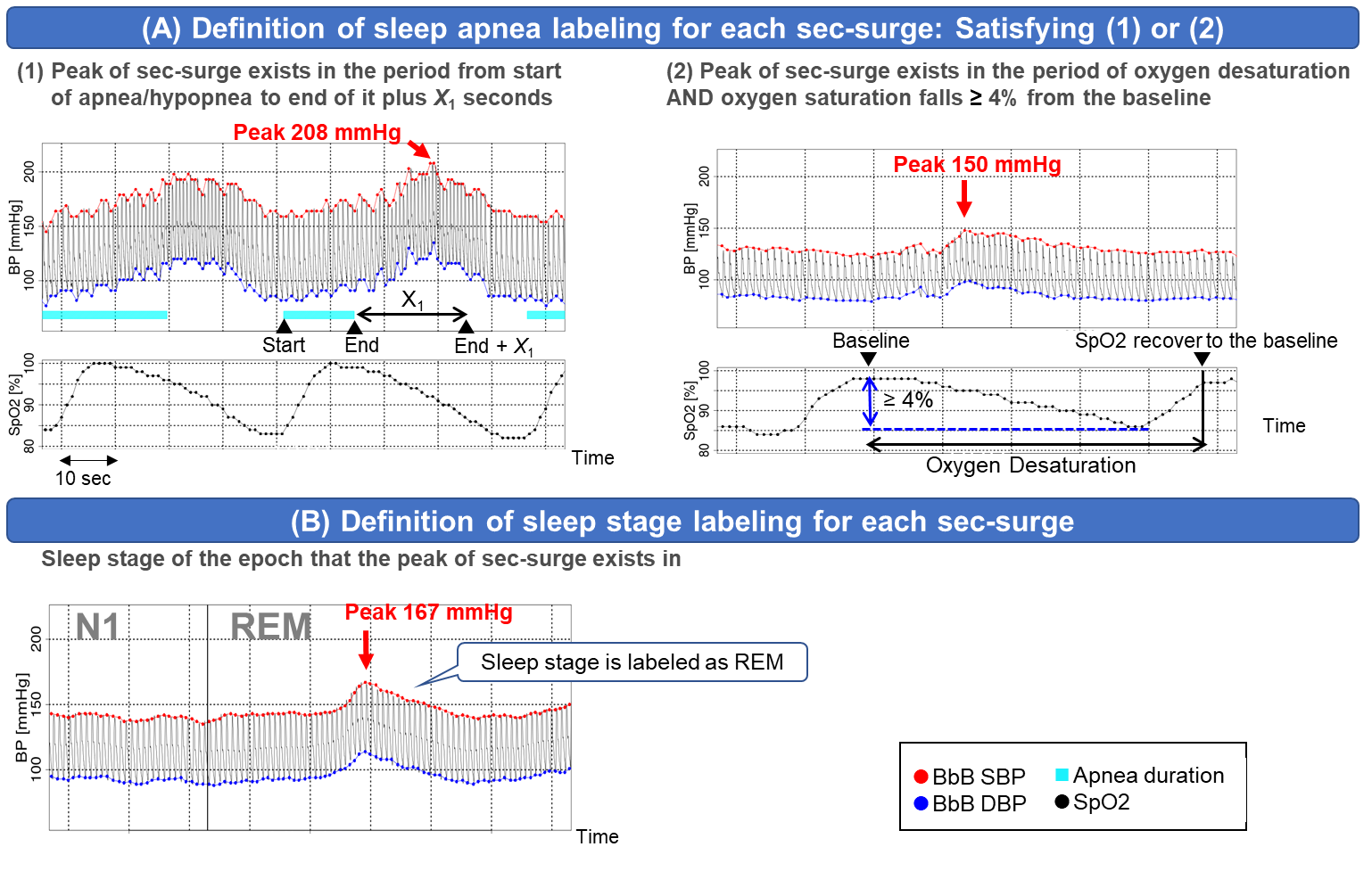


**Supplementary Figure S3.** Labeling rules of sleep apnea and sleep stages to each sec-surge. (A) indicates the definition of sleep apnea labeling. The value of X1 was determined by the distribution of upward duration of sec-surge using 95th percentiles. (B) indicates the definition of sleep stage labeling. BbB indicates beat-by-beat; SBP, systolic blood pressure; DBP, diastolic blood pressure; sec-surge, blood pressure surge in seconds; PSG, polysomnography; and REM, rapid eye movement.


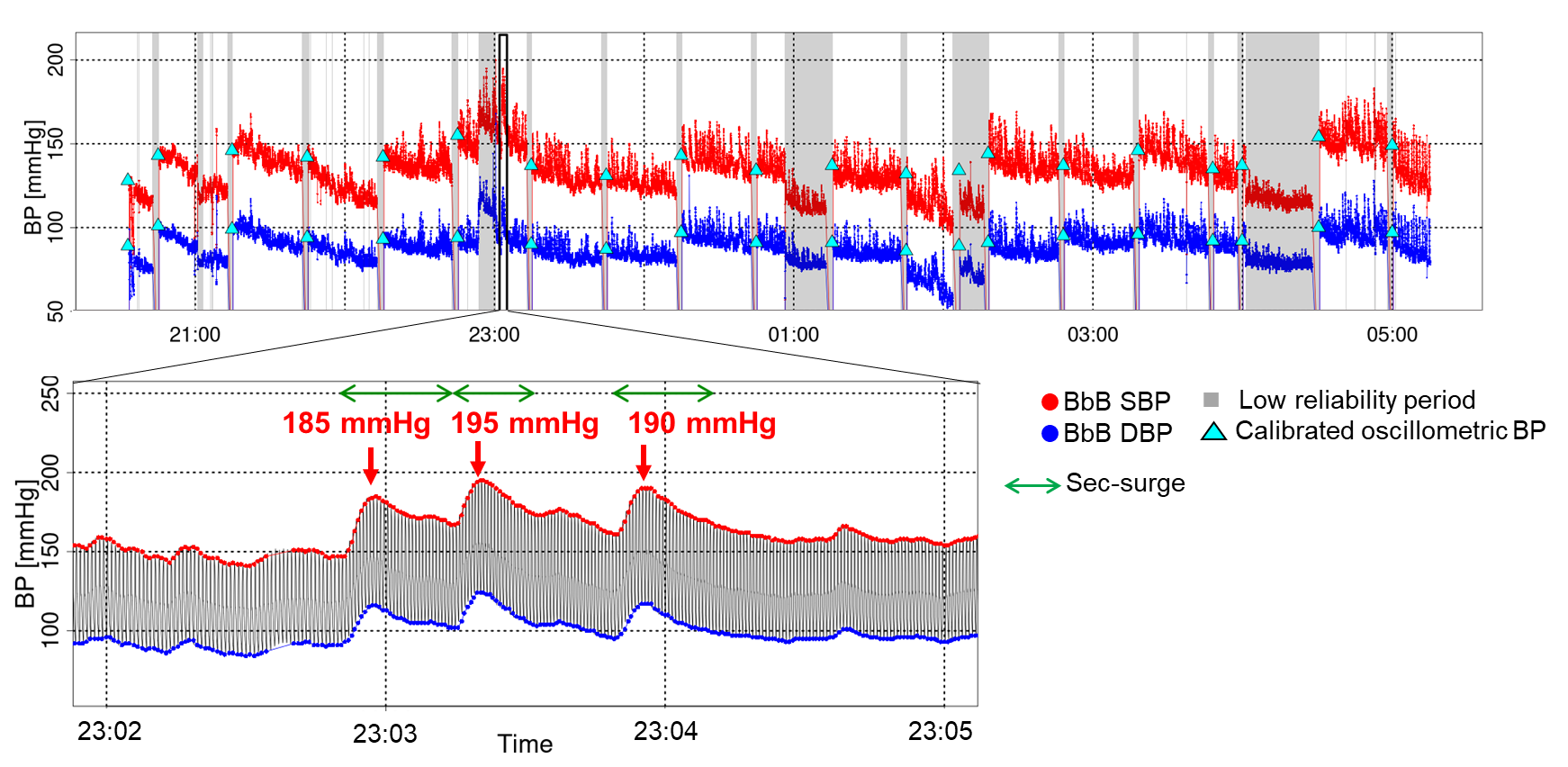


**Supplementary Figure S4.** A typical case of overnight BbB BP measurement and observed sec-surges at night.
Sec-surge indicates blood pressure surge in seconds; BbB, beat-by-beat; SBP, systolic blood pressure; and DBP, diastolic blood pressure.


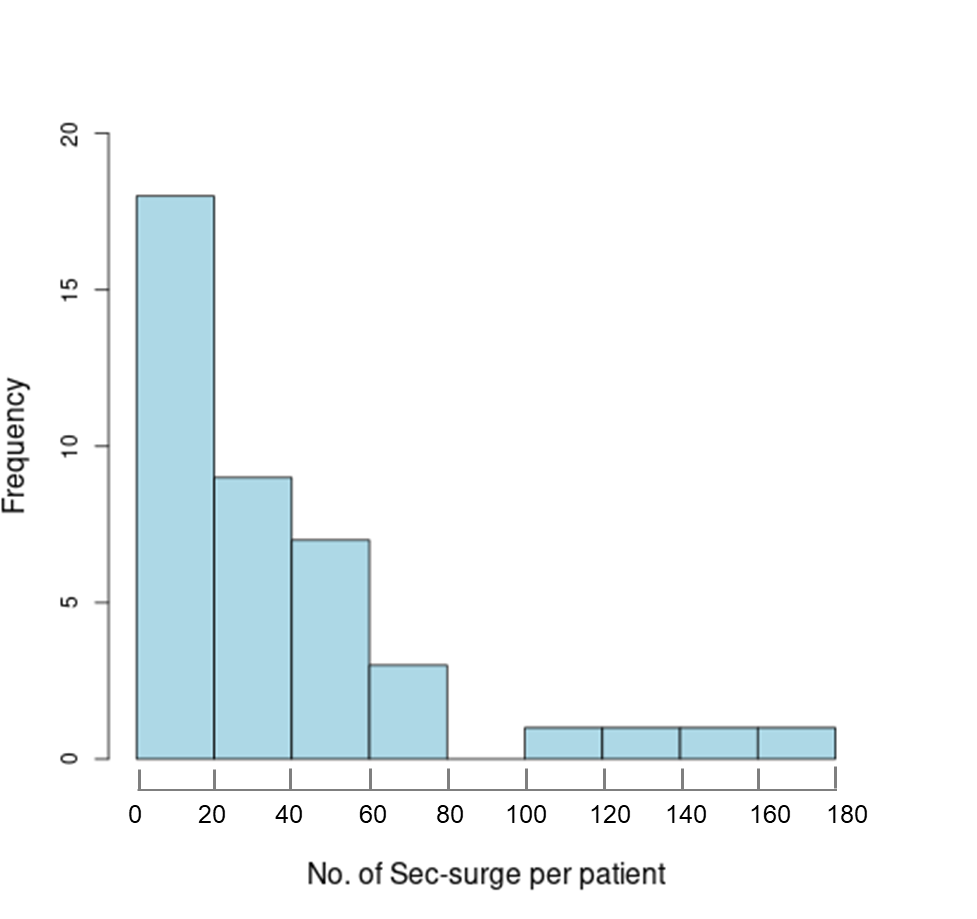


**Supplementary Figure S5.** Histogram of number of sec-surges per patient. Sec-surge indicates blood pressure surge in seconds.


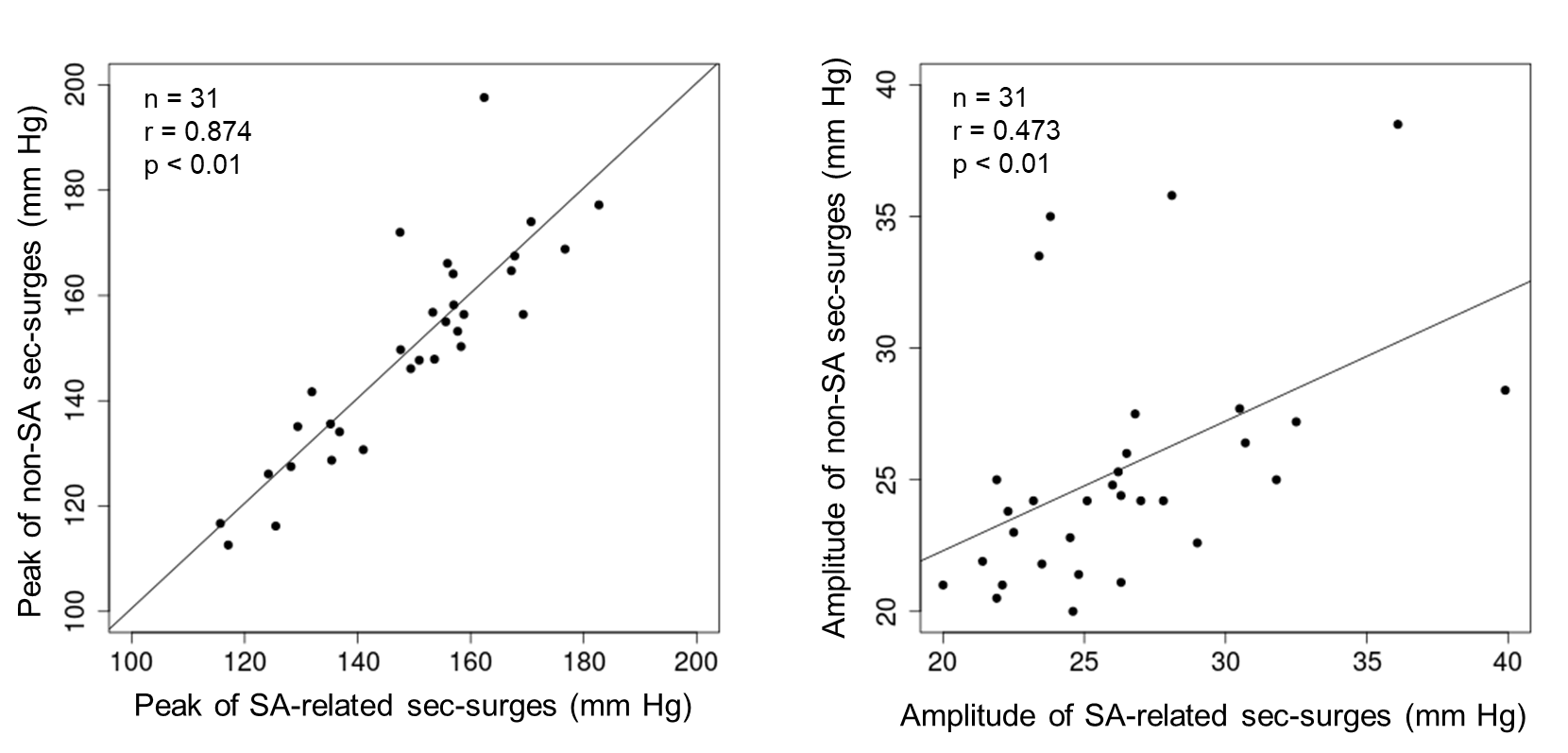


**Supplementary Figure S6.** Association between SA-related sec-surges and non-SA-related sec-surges. Five out of 41 subjects did not have any sec-surges induced by SA, and another five subjects did not have any sec-surges induced by non-SA factors (31 subjects were plotted). The solid line indicates a regression line between SA-related sec-surges and non-SA sec-surges.
Sec-surge indicates blood pressure surge in seconds. SA, sleep apnea
